# The intracellular domain of cell adhesion molecule 1 is present in emphysematous lungs and induces lung epithelial cell apoptosis

**DOI:** 10.1186/s12929-015-0173-8

**Published:** 2015-08-11

**Authors:** Man Hagiyama, Azusa Yoneshige, Takao Inoue, Yasufumi Sato, Takahiro Mimae, Morihito Okada, Akihiko Ito

**Affiliations:** Department of Pathology, Faculty of Medicine, Kinki University, Osaka, 589-8511 Japan; Department of Surgical Oncology, Research Institute for Radiation Biology and Medicine, Hiroshima University, Hiroshima, Japan

**Keywords:** Mitochondrial apoptosis pathway, Protein transfection, γ-secretase, Shedding, Tumor suppressor in lung cancer 1 (TSLC1), Nectin-like molecule 2 (Necl-2)

## Abstract

**Background:**

Pulmonary emphysema is characterized histologically by destruction of alveolar walls and enlargement of air spaces due to lung epithelial cell apoptosis. Cell adhesion molecule 1 (CADM1) is an immunoglobulin superfamily member expressed in lung epithelial cells. CADM1 generates a membrane-associated C-terminal fragment, αCTF, through A disintegrin- and metalloprotease-10-mediated ectodomain shedding, subsequently releasing the intracellular domain (ICD) through γ-secretase-mediated intramembrane shedding of αCTF. αCTF localizes to mitochondria and induces apoptosis in lung epithelial cells. αCTF contributes to the development and progression of emphysema as a consequence of increased CADM1 ectodomain shedding. The purpose of this study was to examine whether the ICD makes a similar contribution.

**Results:**

The ICD was synthesized as a 51-amino acid peptide, and its mutant was synthesized by substituting seven amino acids and deleting two amino acids. These peptides were labeled with fluorescein isothiocyanate and were introduced into various cell lines. ICD peptide-derived fluorescence was well visualized in lung epithelial cells at the site of Mitotracker mitochondrial labeling, but was detected in locations other than mitochondria in other cell types. Mutant peptide-derived fluorescence was detected in locations other than mitochondria, even in lung epithelial cells. Terminal deoxynucleotidyl transferase-mediated dUTP nick-end labeling assays revealed that transduction of the ICD peptide increased the proportion of apoptotic cells 2- to 5-fold in the lung epithelial cell lines, whereas the mutant peptide did not. Abundance of the ICD was below the Western blot detection limit in emphysematous (*n* = 4) and control (*n* = 4) human lungs. However, the ICD was detected only in emphysematous lungs when it was immunoprecipitated with anti-CADM1 antibody (4/4 vs. 0/4, *P* = 0.029).

**Conclusions:**

As the abundance of ICD molecules was sparse but present, increased CADM1 shedding appeared to contribute to the development of emphysema by generating αCTF and the ICD in lung epithelial cells.

**Electronic supplementary material:**

The online version of this article (doi:10.1186/s12929-015-0173-8) contains supplementary material, which is available to authorized users.

## Background

Pulmonary emphysema is a representative chronic obstructive pulmonary disease characterized by destruction of alveolar walls and enlargement of air spaces [1]. These histological characteristics evolve from alveolar and bronchiolar epithelial cell apoptosis and a local imbalance in protease over anti-protease activities [2, 3]. We recently found a molecular link between these two events by analyzing lung epithelial cell adhesion molecule 1 (CADM1), also known as tumor suppressor in lung cancer 1 (TSLC1) and nectin-like molecule 2 (Necl-2) [4]. CADM1 is an intercellular adhesion molecule in the immunoglobulin superfamily. This membrane-spanning glycoprotein is composed of three extracellular Ig-like domains, a single transmembrane region, and a short carboxy-terminal intracytoplasmic tail with a protein 4.1 interaction sequence (P4.1-IS) and a PDZ type II domain-binding motif (PDZ-BM) [5]. The major cell types that express CADM1 in the peripheral lung are bronchiolar and alveolar epithelial cells, but parenchymal lung mast cells and fibroblasts also express it [6, 7]. CADM1 mediates not only lung epithelial cell-cell adhesion, but also lung mast cell adhesion to airway smooth muscle cells [6]. There are several splice isoforms of CADM1; the major ones are named SP1 to 4 [8]. The SP1 isoform reduces survival in HMC-1 mast cells and increases caspase 3/7 activity, whereas the SP4 isoform enhances survival and reduces caspase activity [9]. Recent studies show that CADM1 expression is regulated by post-transcriptional mechanisms, including glycosylation and proteolytic cleavage, referred to as shedding [10, 11]. CADM1 is cleaved at one of two sites in its ectodomain, yielding two membrane-associated C-terminal fragments, αCTF and βCTF [4]. We found that CADM1 shedding increases in emphysematous lungs, and αCTF contributes to apoptosis of lung epithelial cells by localizing in mitochondria [4]. A mutant form of αCTF (αCTFmut) carrying amino acid substitutions and deletions in the intervening region between the P4.1-IS and the PDZ-BM did not localize to mitochondria, suggesting that the intervening region may carry the mitochondrial localization signal [4].

We showed previously that CADM1 αCTF is further cleaved by γ-secretase, an intramembrane-cleaving aspartyl protease, releasing the intracellular domain (ICD) into the cytosol [11]. Based on cleavage site specificity for γ-secretase, CADM1-ICD (C-ICD) is presumed to be a 51-amino acid peptide composed of the full-length intracytoplasmic region (47 amino acids) with four amino acids extending into the transmembrane region toward the N-terminus [11]. It is rather difficult to detect the presence of the C-ICD, probably because it is degraded rapidly, and many other ICD fragments generated by γ-secretase, including the ICD of Notch, a cell membrane-spanning receptor, are degraded rapidly in a proteasome-dependent manner [12, 13]. Despite its low abundance, the Notch-ICD is important for transduction of intracellular signaling mediated by Notch [13].

The purpose of this study was to understand the biomedical relevance of the C-ICD. We first synthesized the C-ICD (51-amino acid peptide) and its mutant form (C-ICDmut; 49-amino acid peptide) carrying the same mutation as that of αCTFmut. We introduced the peptides into various cell lines including lung epithelial cells and examined whether the C-ICD localized to mitochondria and induced cell apoptosis. We next examined whether the C-ICD was present in emphysematous and control human lungs. The results showed that the C-ICD appeared to have actions similar to those of αCTF in lung epithelial cells, thus contributing to the development of emphysema.

## Methods

### Cells

NCI-H441 cells (lot no. 58294188), a human lung epithelial cell line with characteristics of Clara cells, and RLE-6TN cells (lot no. 59111690), a rat lung epithelial cell line with characteristics of alveolar type II cells, were purchased from the American Type Culture Collection (Rockville, MD, USA) in 2010 and 2013, respectively. All experiments using these cells were performed within 4 months after resuscitation. NCI-H441 cells were grown as described in our previous report [4]. RLE-6TN cells were grown in Ham’s F12 medium containing 2 mM L-glutamine (Gibco, Carlsbad, CA, USA) supplemented with 10 % fetal bovine serum, 10 μg/ml bovine pituitary extract (PromoCell, Heidelberg, Germany), 5 μg/ml insulin (Gibco), 2.5 ng/ml insulin-like growth factor (Sigma-Aldrich, St. Louis, MO, USA), 1.25 μg/ml transferrin (Gibco), and 2.5 ng/ml epidermal growth factor (Sigma-Aldrich, St. Louis, MO, USA). COS7 and NIH3T3 cells were described previously [14, 15]. COS7 cells that overexpressed CADM1 exogenously were described previously [11].

### Antibodies and reagents

A rabbit anti-CADM1 polyclonal antibody directed against the C-terminal 15-amino acid peptide was generated in our laboratory as described previously [16]. Other primary antibodies targeting fluorescein isothiocyanate (FITC) (Ab19224; Abcam, Cambridge, UK) and β-actin (mouse monoclonal AC-15; Sigma-Aldrich) were used. Peroxidase- and fluorophore-conjugated secondary antibodies were obtained from Amersham (Buckinghamshire, UK) and Jackson ImmunoResearch (West Grove, PA, USA), respectively. 4’,6’-Diamidino-2-phenylindole (DAPI; Dojindo, Kumakoto, Japan) and silver stain reagents (Sigma-Aldrich) were used according to the manufacturer’s instructions. Phorbol 12-myristate 13-acetate (PMA) was used as described previously [11].

### Peptide synthesis and FITC labeling

The C-ICD and C-ICDmut peptides were synthesized by Fmoc chemistry using the PSSM-8 automated peptide synthesizer (Shimadzu, Kyoto, Japan) and purified by reverse-phase high-performance liquid chromatography on a C18 column with a linear gradient from 0 to 90 % CH_3_CN in 0.1 % trifluoroacetic acid for 60 min at a flow rate of 1 ml/min. The purified peptides were digested with trypsin and separated using the Smart System (GE Healthcare, Little Chalfont, UK) on a RP300 column with a linear gradient from 0 to 90 % CH_3_CN in 0.1 % trifluoroacetic acid for 50 min at a flow rate of 1 ml/min, and the fractions were collected. The individual fractions were analyzed by matrix-assisted laser desorption/ionization time-of-flight collision-induced dissociation tandem mass spectroscopy using the Axima Performance system (Shimadzu), and their amino acid sequences were confirmed by the 492HT protein sequencer (Applied Biosystems, Foster City, CA, USA). FITC-I (Dojindo) was used to label the peptides according to the manufacturer’s instructions. Briefly, the peptide and FITC-I were mixed at a weight ratio of 10:1 in phosphate-buffered saline (PBS) containing 2.5 mM carbonate and were reacted at 4 °C for 4 h. The FITC-labeled peptide was washed with PBS by five centrifugation cycles using an Amicon Ultra-10 K membrane filter (Millipore, Billerica, MA, USA).

### Peptide transduction

The Xfect Protein Transfection kit (Takara Bio Inc., Shiga, Japan) was used to introduce the peptides into cells according to the manufacturer’s instructions. Briefly, cells were grown to 60–70 % confluence in coverslip-like-bottomed 35-mm diameter culture dishes (μ-Dishes; ibidi, Verona, WI, USA) and incubated at 4 °C for 1 h in serum-free medium supplemented with 0.25 × Xfect protein transfection reagent containing 0.4 μg (for RLE-6TN cells) or 0.2 μg (for the other cells) of either the C-ICD or C-ICDmut peptide labeled with FITC or unlabeled, as indicated. The cells were washed with serum-free medium and incubated in growth medium. The same treatment was applied 24 h later. The cells were subjected to Western blot analyses after an additional 24 h, and mitochondrial labeling or terminal deoxynucleotidyl transferase-mediated dUTP nick end labeling (TUNEL) assays were conducted.

### TUNEL assay

The TUNEL assay was performed using NCI-H441 and RLE-6TN cells and the In Situ Cell Death Detection kit (Roche Applied Science, Upper Bavarie, Germany) according to the manufacturer’s instructions [4]. Briefly, the cells were fixed with 4 % paraformaldehyde, permeabilized with 0.1 % Triton X-100 in 0.1 % sodium citrate (pH 7.4), and incubated with the TUNEL reaction mixture containing terminal deoxynucleotidyl transferase and FITC-labeled dUTP for 1 h at 37 °C, followed by nuclear counterstaining with DAPI. The double-stained cells were observed through a fluorescence microscope (Axioskop 2 plus; Carl Zeiss, Oberkochen, Germany). A cell was deemed TUNEL-positive if it exhibited TUNEL signals among the DAPI nuclear stain. The number of TUNEL-positive cells was counted among 500 cells. In some experiments, FITC-labeled peptide-transfected NCI-H441 and RLE-6TN cells were analyzed with a Click-iT Plus TUNEL Assay Kit containing an Alexa Flour 594-conjugated secondary antibody (Molecular Probes, Eugene, OR, USA), as we described previously [17]. The number of TUNEL-positive cells was counted among 300 cells either FITC-positive or -negative. All measurements were performed in triplicate, and the mean and standard error of the proportion of TUNEL-positive cells were calculated for each experimental group. The TUNEL assays were repeated three times with similar results.

### Mitochondrial labeling

Mitochondria were labeled with red fluorescence as described previously [4]. Briefly, cells were incubated in growth medium containing 200 nM Mitotracker (Molecular Probes, Eugene, OR, USA) for 30 min and washed with PBS twice. Then, images of the stained cells were captured using a confocal laser microscope (LSM510 Meta; Carl Zeiss).

### Human samples

Human lung tissues were obtained from patients diagnosed with lung cancer who underwent pulmonary lobectomy or segmentectomy at Hiroshima University Hospital (Hiroshima, Japan) between 2010 and 2013. All smokers were obliged to quit smoking >1 month before the surgery. Non-cancerous portions (approximately 2 cm^3^) of the surgical specimens were cut into two pieces immediately after the operation; one was fixed in 10 % buffered formalin to prepare hematoxylin and eosin (H&E)-stained tissue sections, and the other was frozen to prepare lung tissue lysates. When an H&E-stained specimen was consistently diagnosed as emphysematous by two pathologists, the patient was included in the present study as an “emphysematous lung” case. In contrast, when the two pathologists consistently detected few pathological changes in an H&E-stained specimen, the patient was included as a “normal lung” case. All patients provided written informed consent to participate in this study, and our institutional review board approved the experimental protocol (approval number, Eki-350).

### Western blot analysis and immunoprecipitation

Cultured cell pellets and frozen lung tissues were lysed in Tris-buffered saline (pH 7.4) containing 1 % sodium dodecyl sulfate, 5 mM ethylenediaminetetraacetic acid, and a protease inhibitor cocktail (Sigma-Aldrich). Insoluble components were removed by centrifugation, and the supernatant was used as a cell or lung tissue lysate. Western blot analyses were conducted, and immunoreactive band intensities were quantified using NIH ImageJ software, as described previously [18]. The lung lysates were diluted with PBS (1:9, v/v) for immunoprecipitation, prior to adding the anti-CADM1 antibody. The procedures were essentially the same as described in our past report [19].

### Rat primary cultured alveolar epithelial cells (AECs)

Type 1 AECs were isolated from Sprague–Dawley rats, and were cultured to differentiate into type 2 AECs according to the published protocol [20]. Experiments with rats were performed in accordance with the Guide for Animal Experimentation of Kinki University (approval number, KAME-25-019).

### Reverse transcription (RT)-PCR

The methods of RNA extraction, reverse transcription, and PCR to detect CADM1 isoforms were essentially identical to those described previously [16]. PCR primers for caveolin-1, a type 1 AEC marker, and surfactant protein B, a type 2 AEC marker, were according to past studies [21, 22].

### Statistical analysis

The proportions of TUNEL-positive cells were compared between the two groups using the Student’s *t*-test. The number of patients expressing the C-ICD in lungs was assessed between the two groups using Fisher’s exact test. A *P*-value ≤ 0.05 was considered significant.

## Results

### The C-ICD peptide localizes to mitochondria and induces apoptosis in lung epithelial cells

**Fig. 1:**
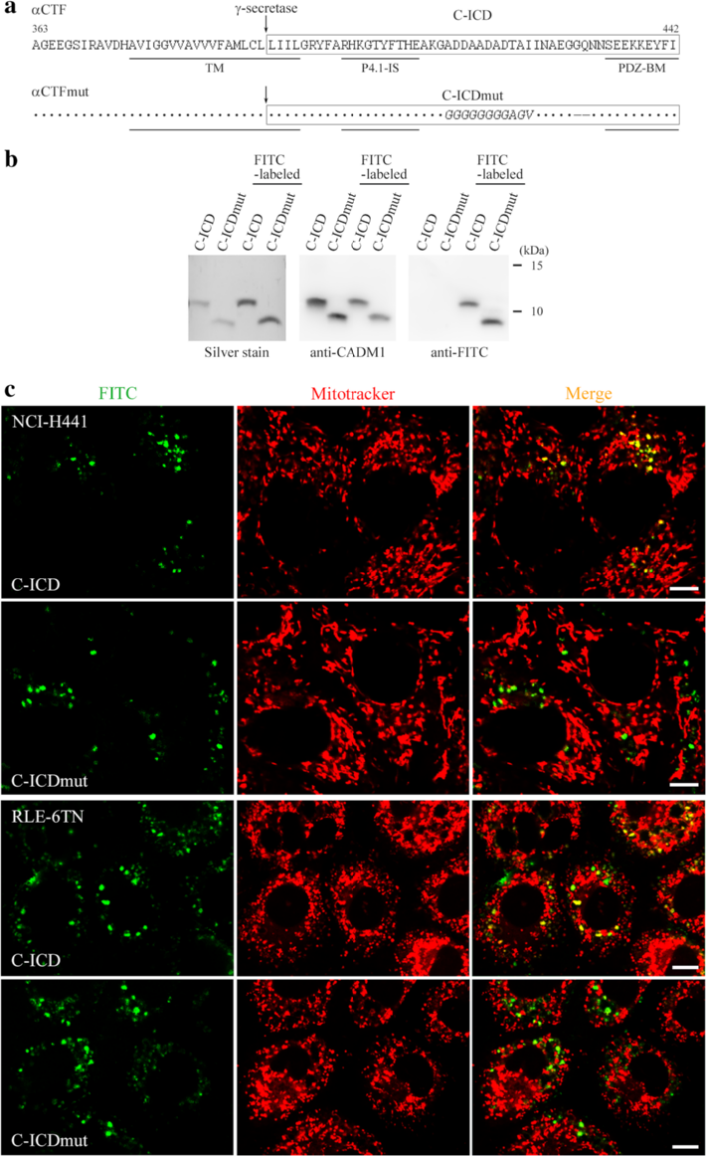
Mitochondrial localization of the cell adhesion molecule 1 intracellular domain (C-ICD) peptide in lung epithelial cell lines. **a** Full-length amino acid sequences of the α-C-terminal fragment (αCTF) and the αC-terminal fragment mutant (αCTFmut) of cell adhesion molecule 1. The C-ICD and the cell adhesion molecule 1 intracellular domain mutant (C-ICDmut) are shown in the boxed portions. The arrow indicates the γ-secretase cleavage site. Dots indicate the amino acid residues as above. The 7-amino acid substitution and the 2-amino acid deletion in C-ICDmut are shown in italics and –, respectively. The structural motifs are underlined. TM, transmembrane domain; P4.1-IS, protein 4.1 interaction sequence; PDZ-BM, PDZ type II domain-binding motif. Amino acid residues are numbered according to the NCBI database sequence (accession number, CCD32610). **b** The unlabeled and FITC-labeled C-ICD and C-ICDmut peptides were detected by sodium dodecyl sulfate polyacrylamide gel electrophoresis, and by silver staining (left) or Western blotting of the gels using antibodies against cell adhesion molecule 1 (CADM1) (middle) or FITC (right). **c** NCI-H441 (upper) and RLE-6TN (lower) cells were introduced with the FITC-labeled C-ICD or C-ICDmut peptide and stained with Mitotracker. Green (FITC) and red (Mitotracker) fluorescent images were merged, as shown in the right panels. Bar = 10 μm

We previously detected the C-ICD in lysates of COS7 cells that had been modified to overexpress CADM1, and we reported a presumed γ-secretase αCTF cleavage site [11]. According to the cleavage site, we chemically synthesized C-ICD as a 51-amino acid peptide. We also synthesized a 49-amino acid peptide (C-ICDmut) as a control by using the same cleavage site as αCTFmut, which carried seven amino acid substitutions and two amino acid deletions (Fig. 1a) [4]. Successful synthesis was confirmed by LC-MS/MS (Additional file 1: Figure S1). The C-ICD and C-ICDmut peptides were detected at approximately 11 and 9 kDa on a Western blot, respectively (Fig. 1b), although the theoretical molecular weight of the C-ICD peptide is 5.3 kDa [11] (Fig. 1b). These peptides were introduced into NCI-H441 and RLE-6TN lung epithelial cells using Xfect reagents after FITC labeling. As peptide transduction efficacy was rather low, we conducted the Xfect treatment on identical cell cultures once daily for two consecutive days and stained the cells with Mitotracker dye, a mitochondrial marker, the next day. The FITC-labeled C-ICD and C-ICDmut peptides were both detected as fine or coarse aggregates in the cytoplasm (Fig. 1c, left). Most of the C-ICD green signals turned yellow when the FITC and Mitotracker fluorescent images were merged, whereas the C-ICDmut signals remained green, indicating that the C-ICD signals preferentially colocalized with Mitotracker stain, whereas the C-ICDmut signals were not (Fig. 1c, right). Peptide transduction experiments were conducted on COS7 and NIH3T3 cells. The FITC-labeled C-ICD and C-ICDmut peptides were detected as fine or coarse aggregates in the cytoplasm but did not colocalize with the Mitotracker stain (Additional file 2: Figure S2).

**Fig. 2:**
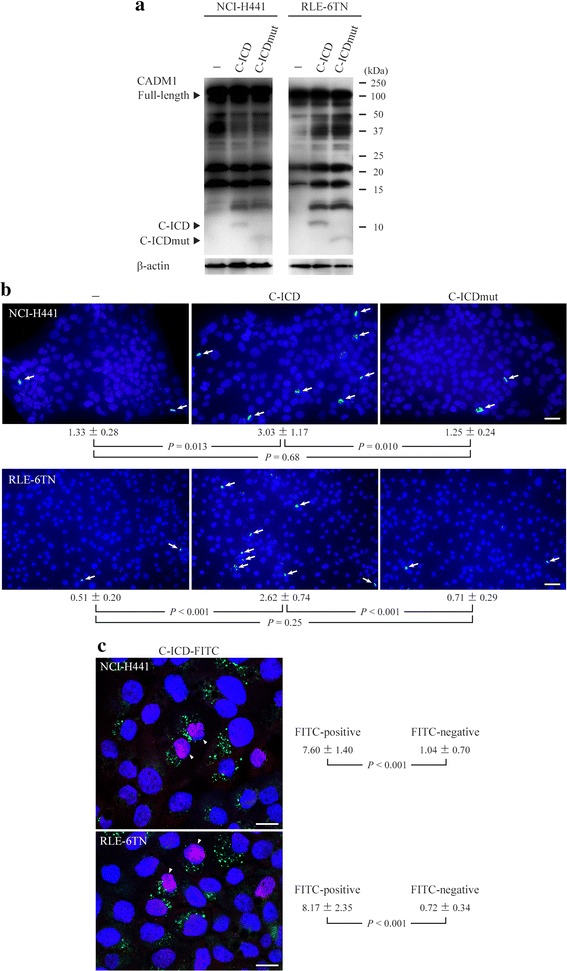
Induction of lung epithelial cell apoptosis by the cell adhesion molecule 1 intracellular domain (C-ICD) peptide. NCI-H441 and RLE-6TN cells were treated with Xfect solutions containing the unlabeled C-ICD or the cell adhesion molecule 1 intracellular domain mutant (C-ICDmut) peptide or were left untreated (−). The cells were analyzed the next day by Western blotting using anti-cell adhesion molecule 1 (CADM1) antibody (**a**). Arrowheads indicate immunoreactive bands corresponding to full-length CADM1, the C-ICD. The blots were reprobed with anti-β-actin antibody to indicate protein loading. Another set of cells was analyzed by terminal deoxynucleotidyl transferase-mediated dUTP nick end labeling (TUNEL) assay (**b**). The mean proportions of TUNEL-positive cells (depicted by arrows) and standard errors are indicated under the panels. The *P*-values were determined by Student s *t*-test. Bar = 50 μm. **c** Similar TUNEL assays were done using the FITC-labeled C-ICD peptide (C-ICD-FITC). The mean proportions of TUNEL-positive cells and standard errors were calculated in each of FITC-positive and -negative cells. Cells double positive for FITC and TUNEL are depicted by arrowheads. The *P*-values were determined by Student’s *t*-test. Bar = 20 μm

The C-ICD may induce apoptosis of lung epithelial cells by accumulating in mitochondria, as seen with αCTF [4]. To test this possibility, we used peptides without FITC labeling to prevent FITC from masking the proper actions of the peptides. The day after two Xfect treatments over two consecutive days, we analyzed the cells by Western blot analysis and TUNEL assays. Western blot analyses weakly but successfully detected the C-ICD and C-ICDmut peptides at roughly equal intensities in NCI-H441 and RLE-6TN cells (Fig. 2a). The proportions of TUNEL-positive cells were approximately 1 % in both cell lines when left untreated or when introduced with the C-ICDmut peptide, whereas the proportions increased 2.3- and 5.1-fold in NCI-H441 and RLE-6TN cells, respectively, when introduced with the C-ICD peptide (Fig. 2b). Similar TUNEL assays were done on NCI-H441 and RLE-6TN cells using the FITC-labeled C-ICD peptide. The proportion of TUNEL-positive cells reached to 7.8 % in NCI-H441 cells positive for FITC (*i.e.*, cells containing the C-ICD peptide), whereas it was below 1 % in FITC-negative cells (*i.e.*, cells not containing the C-ICD peptide). Similar results were obtained with RLE-6TN cells (Fig. 2c).

### The abundance of C-ICD molecules is sparse but present in emphysematous lungs

**Fig. 3:**
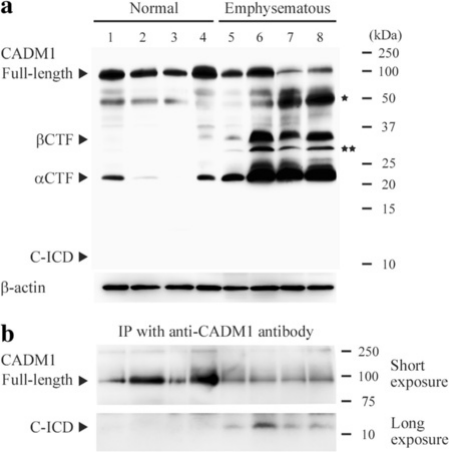
Detection of the cell adhesion molecule 1 intracellular domain (C-ICD) in emphysematous lungs. **a** Western blot analysis of cell adhesion molecule 1 (CADM1) expression in normal and emphysematous lungs. Cases are numbered as in Table 1. Arrowheads indicate the immunoreactive bands corresponding to full-length CADM1, the β-C-terminal fragment (βCTF), the αCTF, and the C-ICD. Bands corresponding to the non-glycosylated full-length form and βCTF are depicted by one and two asterisks, respectively. The blot was reprobed with anti-β-actin antibody to indicate protein loading. **b** The lung lysates were subjected to immunoprecipitation using anti-CADM1 antibody and to Western blotting using the same antibody. Immunoreactive bands were detected after short and longer exposure times

We obtained histologically normal (*n* = 4) and emphysematous (*n* = 4) lung tissues from patients who underwent lung lobectomy due to cancer. The patient characteristics are summarized in Table 1, and the lung histology is shown in Additional file 3: Figure S3. The tissue lysates were Western blotted using the CADM1 antibody. Consistent with our previous report [4], the quantity of αCTF relative to full-length CADM1 increased in emphysematous lungs, but the quantity of C-ICD was below the detection limit (Fig. 3a). The lung lysates were then subjected to immunoprecipitation using the CADM1 antibody and Western blotting using the same antibody. All eight samples expressed full-length CADM1 as abundantly as in the original lysates, whereas the C-ICD was essentially undetectable in four normal lungs but certainly detectable in all four emphysematous lungs (Fig. 3b; 4/4 vs. 0/4, *P* = 0.029). To confirm the molecular weight of the C-ICD, we used COS7 cells that overexpressed CADM1 exogenously, because we previously detected the C-ICD in the lysate of these cells by treatment with PMA, a shedding inducer [11]. Western blot analyses revealed that the C-ICD peptide was identical in molecular weight (approximately 11 kDa) to the C-ICD from the COS7 cell lysate (Additional file 4: Figure S4).

**Table 1:** Clinical characteristics of patients with normal and emphysematous lungs

	Normal				Emphysematous			
Case	1	2	3	4	5	6	7	8
Age	60	48	72	74	58	71	71	87
Sex	M	M	F	F	M	F	F	M
Brinkman index	800	0	0	0	370	0	0	2800
Cause of surgery^a^	SQ	AD	AD	AD	AD	AD	AD	SQ
Excised lung lobe^b^	LU	LL	RU	LL	LL	LU	RU	RU
FEV1/FVC	NE	83.1	74.6	71.2	77.3	73.4	80.5	71.5
DLCO (%)^c^	NE	NE	NE	NE	NE	NE	NE	57.6

^a^ *AD* adenocarcinoma, *SQ* squamous cell carcinoma

^b^ *RU* right upper, *RL* right lower, *LU* left upper, *LL* left lower

^c^ *NE* not examined

### Detection of CADM1 isoforms expressed in lung epithelial cells

**Fig. 4:**
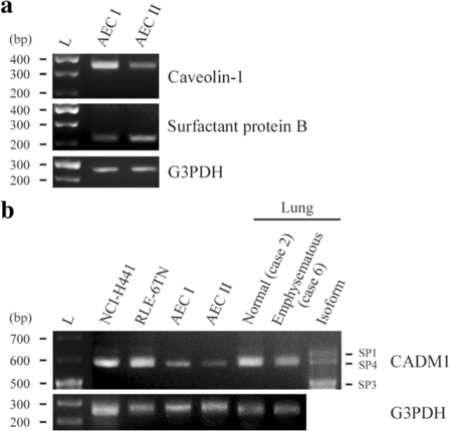
CADM1 isoforms expressed in human lungs and lung epithelial cells. Total RNAs were extracted from rat primary cultured type 1 and type 2 AECs, and were analyzed by RT-PCR using primer sets for calveolon-1 and surfactant protein B (**a**). Total RNAs from NCI-H441 and RLE-6TN human lung epithelial cell lines, rat type 1 and type 2 AECs and human lung tissues of cases indicated were analyzed by RT-PCR using a primer set encompassing the CADM1 extracellular juxtamembrane region, susceptible to alternative splicing (**b**). The PCR products were electrophoresed on 3 % agarose gels, together with CADM1 isoform size markers (rightmost lane in B). L, 100 base pair (bp) ladder. RNAs were also PCR-amplified using a primer set for G3PDH to indicate RNA loading per lane

Because individual CADM1 isoforms have different properties [9], it is significant to determine which isoforms are expressed in lung epithelial cells. We isolated type 2 AECs from the rat lung, and cultured them to differentiate into type 1 AECs. Successful preparation of these primary cells was verified by RT-PCR analyses showing that type 1 and type 2 AECs had more mRNA for their corresponding marker proteins, caveolin-1 [21] and surfactant protein B [22] (Fig. 4a). RT-PCR analyses to detect CADM1 mRNA revealed that SP4 was exclusively expressed in type 1 and type 2 AECs, two lung epithelial cell lines and human lung tissues examined in the present study (Fig. 4b).

## Discussion

The C-ICD peptide was localized preferentially to mitochondria in NCI-H441 and RLE-6TN cells, but it was occasionally detected elsewhere. In addition, mitochondrial localization of the C-ICD appeared to depend on the cell type, because it was not observed in COS7 or NIH3T3 cells. These results are consistent with our previous notion that αCTF lacks a canonical mitochondrial targeting sequence but is transported to mitochondria by binding to particular chaperones, such as heat shock proteins 40 and 90 [4, 23, 24]. Lung epithelial cells may express functional chaperones that efficiently transport cytosolic molecules to mitochondria.

Localization of the C-ICD in mitochondria appeared to result in the induction of apoptosis. Similar mechanisms have been suggested for other molecules. The adenomatous polyposis coli (APC) tumor suppressor is cleaved by caspase to release an N-terminal 777-amino acid fragment, which localizes to mitochondria and facilitates apoptosis [25, 26]. Transcription factor p53 and activating transcription factor two localize in mitochondria under genotoxic stress and depolarize the outer membrane potential [27–30]. Molecules that accumulate in the mitochondria as an alternative may accelerate already initiated apoptosis. Interestingly, the C-ICD is likely to accumulate in the mitochondrial intermembrane space because it is small enough to pass through the porin pores, in which a voltage-dependent anion channel protein spans the outer membrane [31, 32]. The intermembrane space has attracted attention as an organelle microdomain important for mitochondrial function, because it contains not only executioners of apoptosis, such as Smac and cytochrome *c* [33], but also critical factors for the maintenance of mitochondrial dynamics and homeostasis, such as adenylate kinase and polynucleotide phosphorylase [33, 34]. Accumulation of the C-ICD in this space may perturb mitochondrial integrity and trigger depolarization of the outer membrane potential.

Ectodomain shedding of CADM1 is a prerequisite for generation of C-ICD [11]. Ectodomain cleavability varies among CADM1 isoforms; SP3 is non-cleavable, SP1 and SP2 are constitutively cleavable, and SP4 is inducibly cleavable by pathological stimuli [11, 35]. As shown in Fig. 4, lung epithelial cells appeared to express SP4 exclusively. Therefore, C-ICD may be generated in lung epithelial cells under specific pathological conditions where SP4 ectodomain shedding is induced, such as emphysema and interstitial pneumonia [4, 17].

We showed that C-ICD molecules were sparse but present in emphysematous lungs, suggesting a contribution of this domain to the development of emphysema. This speculation needs detailed evaluation by future experiments using primary human lung epithelial cells and animal models. As the C-ICD is produced by γ-secretase, γ-secretase inhibitors may be effective at halting the progression of lung epithelial apoptosis in patients with emphysema. Various γ-secretase inhibitors have been developed as treatments for Alzheimer’s disease, and some are currently under phase I - III preclinical evaluation [36, 37] and may be applicable to patients with emphysema. Our results deepen the understanding of emphysema pathogenesis and may open a new avenue for target-based therapeutic approaches to the disease.

## Conclusions

The C-ICD localized in mitochondria, induced apoptosis in lung epithelial cells, and was scarce in abundance but present in emphysematous lungs. Therefore, the C-ICD appears to contribute to the development and progression of pulmonary emphysema.

## Additional files

Additional file 1: Figure S1. Synthesis of the cell adhesion molecule 1 intracellular domain (C-ICD) and the mutated cell adhesion molecule 1 intracellular domain (C-ICDmut) peptides. High-performance liquid chromatography data of the synthesized peptides. The C-ICD (upper) and C-ICDmut (lower) peptides were detected at 5657.09 (theoretical value 5657.78) and 5127.55 (theoretical value 5126.57) m/z, respectively. (TIFF 356 kb) Additional file 2: Figure S2. Subcellular localization of the intracellular domain (ICD) and the mutated intracellular domain (ICDmut) peptides in COS7 and NIH3T3 cells. COS7 (left) and NIH3T3 (right) cells were introduced with the FITC-labeled C-ICD (upper) or the C-ICDmut (lower) peptide and stained with Mitotracker. Green (FITC) and red (Mitotracker) fluorescent images were merged. Bar = 10 μm. (TIFF 1683 kb) Additional file 3: Figure S3. Histology of normal and emphysematous lungs. Representative histological images of normal (case no.2) and emphysematous (case no.7) lungs are shown in the left and right panels, respectively. Hematoxylin and eosin stain. Bar = 100 μm. (TIFF 4669 kb) Additional file 4: Figure S4. Detection of the cell adhesion molecule 1 intracellular domain (C-ICD) and C-ICD peptide by Western blot analysis. Original COS7 and CADM1-overexpressing COS7 (COS7-CADM1) cells were left untreated (−) or treated with PMA (200 ng/ml; +). These cell lysates and the solutions containing the C-ICD or mutant C-ICD (C-ICDmut) peptides were analyzed by Western blotting using anti-cell adhesion molecule 1 (CADM1) antibody. Immunoreactive bands were detected after short and longer exposure times. The blot was reprobed with anti-β-actin antibody to indicate protein loading. Arrowheads indicate immunoreactive bands corresponding to the various forms of CADM1 and peptides named. (TIFF 142 kb)

## Abbreviations

C-ICD: Cell adhesion molecule 1 intracellular domain
C-ICDmut: Mutant form of C-ICD
CADM1: Cell adhesion molecule 1
CTF: C-terminal fragment
FITC: Fluorescein isothiocyanate
H&E: Hematoxylin and eosin
ICD: Intracellular domain
P4.1-IS: Protein 4.1 interaction sequence
PDZ-BM: PDZ type II domain-binding motif
TUNEL: Terminal deoxynucleotidyl transferase-mediated dUTP nick end labeling
