# Supplementary figures and images for: The intracellular domain of cell adhesion molecule 1 is present in emphysematous lungs and induces lung epithelial cell apoptosis
Source: J Biomed Sci. 2015 Aug 11;22(1):67. doi: 10.1186/s12929-015-0173-8 (PMC4531499; doi:10.1186/s12929-015-0173-8)

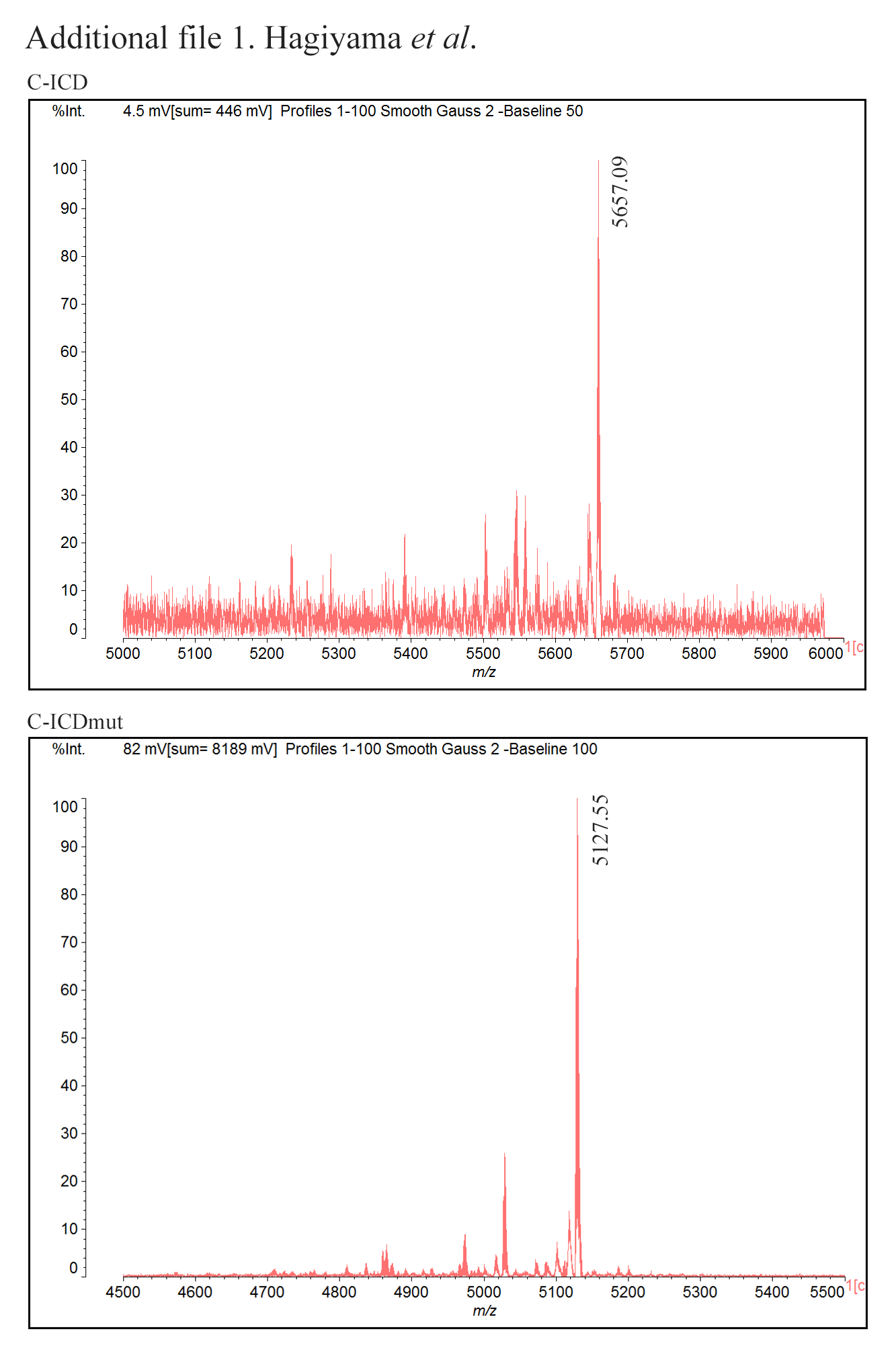

Supplement: Additional file 1: Figure S1. — Synthesis of the cell adhesion molecule 1 intracellular domain (C-ICD) and the mutated cell adhesion molecule 1 intracellular domain (C-ICDmut) peptides. High-performance liquid chromatography data of the synthesized peptides. The C-ICD (upper) and C-ICDmut (lower) peptides were detected at 5657.09 (theoretical value 5657.78) and 5127.55 (theoretical value 5126.57) m/z, respectively. (TIFF 356 kb) [file 12929_2015_173_MOESM1_ESM.tif]

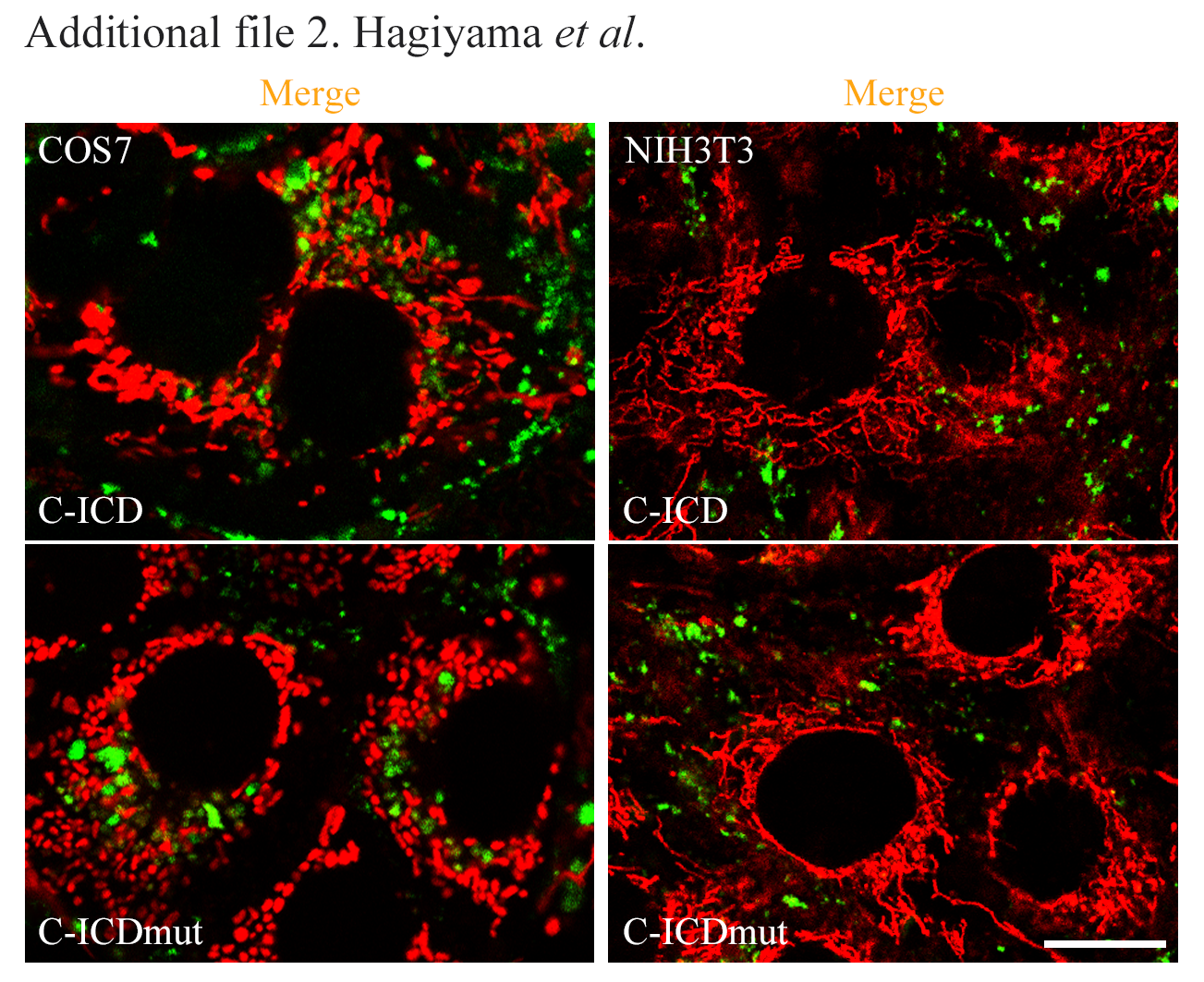

Supplement: Additional file 2: Figure S2. — Subcellular localization of the intracellular domain (ICD) and the mutated intracellular domain (ICDmut) peptides in COS7 and NIH3T3 cells. COS7 (left) and NIH3T3 (right) cells were introduced with the FITC-labeled C-ICD (upper) or the C-ICDmut (lower) peptide and stained with Mitotracker. Green (FITC) and red (Mitotracker) fluorescent images were merged. Bar = 10 μm. (TIFF 1683 kb) [file 12929_2015_173_MOESM2_ESM.tif]

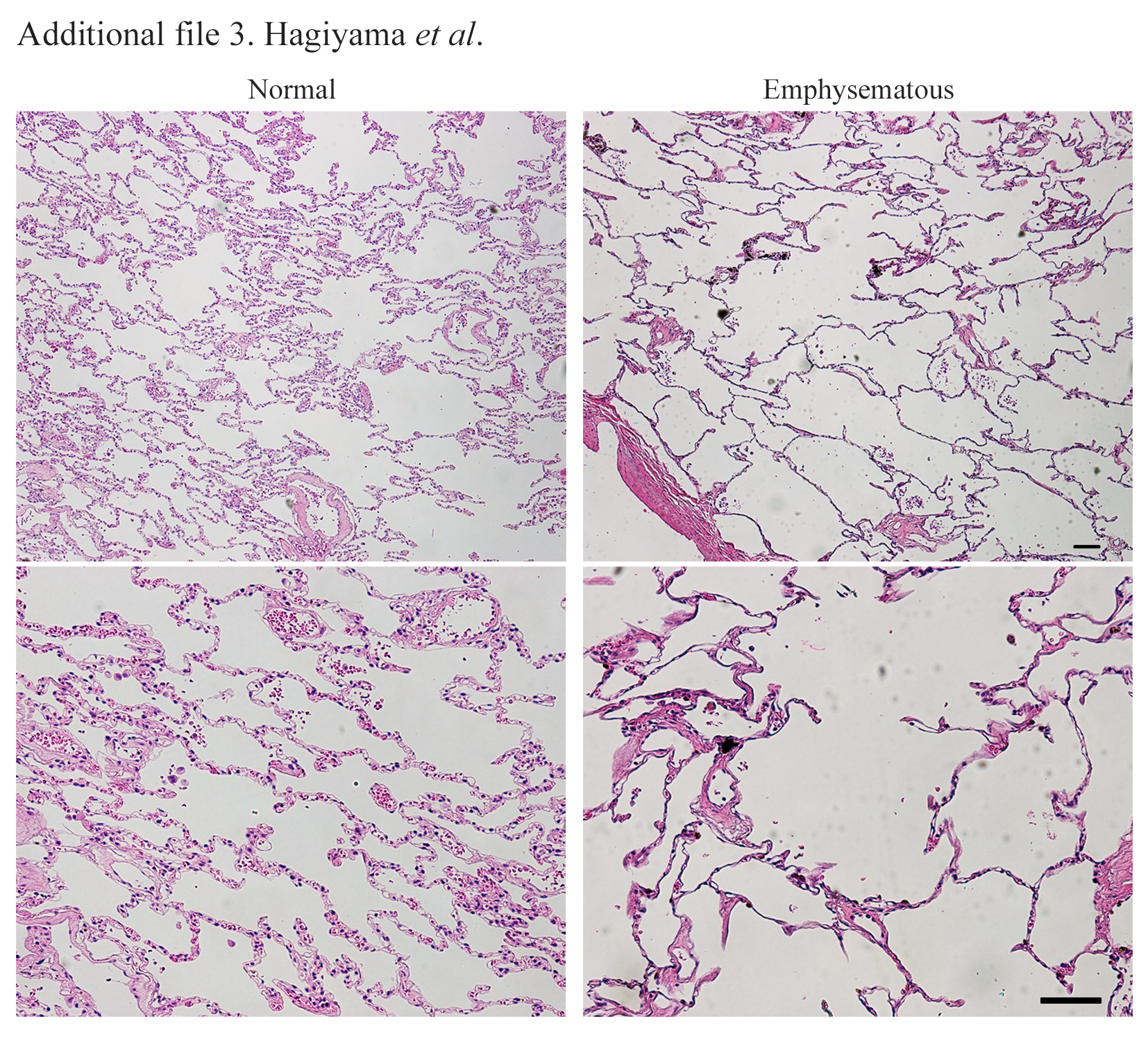

Supplement: Additional file 3: Figure S3. — Histology of normal and emphysematous lungs. Representative histological images of normal (case no.2) and emphysematous (case no.7) lungs are shown in the left and right panels, respectively. Hematoxylin and eosin stain. Bar = 100 μm. (TIFF 4669 kb) [file 12929_2015_173_MOESM3_ESM.tif]
